# A highly active and stable hydrogen evolution catalyst based on pyrite-structured cobalt phosphosulfide

**DOI:** 10.1038/ncomms10771

**Published:** 2016-02-19

**Authors:** Wen Liu, Enyuan Hu, Hong Jiang, Yingjie Xiang, Zhe Weng, Min Li, Qi Fan, Xiqian Yu, Eric I. Altman, Hailiang Wang

**Affiliations:** 1 Department of Chemistry and Energy Sciences Institute, Yale University, 520 West Campus Drive, West Haven, Connecticut 06511, USA; 2 Chemistry Department, Brookhaven National Laboratory, Upton, New York 11973, USA; 3 Beijing National Laboratory for Molecular Sciences, College of Chemistry and Molecular Engineering, Peking University, Beijing 100871, China; 4 Department of Mechanical Engineering and Materials Science, Yale University, 520 West Campus Drive, West Haven, Connecticut 06511, USA; 5 Department of Chemical and Environmental Engineering, Yale University, 520 West Campus Drive, West Haven, Connecticut 06511, USA

## Abstract

Rational design and controlled synthesis of hybrid structures comprising multiple components with distinctive functionalities are an intriguing and challenging approach to materials development for important energy applications like electrocatalytic hydrogen production, where there is a great need for cost effective, active and durable catalyst materials to replace the precious platinum. Here we report a structure design and sequential synthesis of a highly active and stable hydrogen evolution electrocatalyst material based on pyrite-structured cobalt phosphosulfide nanoparticles grown on carbon nanotubes. The three synthetic steps in turn render electrical conductivity, catalytic activity and stability to the material. The hybrid material exhibits superior activity for hydrogen evolution, achieving current densities of 10 mA cm^−2^ and 100 mA cm^−2^ at overpotentials of 48 mV and 109 mV, respectively. Phosphorus substitution is crucial for the chemical stability and catalytic durability of the material, the molecular origins of which are uncovered by X-ray absorption spectroscopy and computational simulation.

## Abstract (web summary)

Rational design and synthesis are important for the development of materials for energy applications. Here, the authors sequentially synthesize pyrite structured cobalt phosphosulfide nanoparticles on carbon nanotubes, probing the role of phosphorous substitution on catalyst stability and durability.

## Graphical abstract

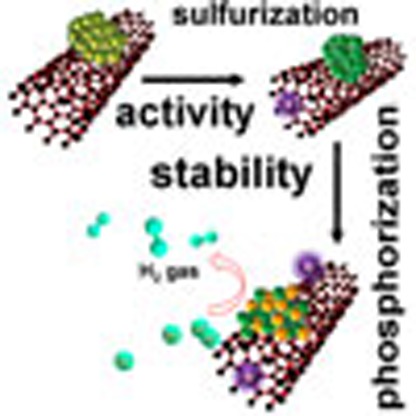

With the rising concern over energy crisis and environmental pollution, there has been a growing need to replace fossil fuels with clean and sustainable energy carriers. Molecular hydrogen, with its high energy density and non-polluting characteristics, has been regarded as one of the most promising new fuels^1^,^2^. There are various methods to produce hydrogen, among which water splitting driven by electricity generated from renewable energy sources is an attractive way to support the future hydrogen economy. Efficient electrolytic hydrogen generation relies heavily on active, durable and affordable catalysts to accelerate the kinetics^3^,^4^,^5^. Although platinum (Pt) has been widely acknowledged as the most active catalyst for the hydrogen evolution reaction (HER), this precious metal is scarce in the earth's crust and expensive for large scale applications.

In pursuit of inexpensive replacements for Pt as the HER electrocatalyst in acidic solutions, numerous inorganic materials based on non-precious transition metals, including sulfides^6^,^7^,^8^, selenides^9^,^10^, phosphides^11^,^12^,^13^ and others^14^,^15^ have been explored. Extensive effort has been devoted to improving the HER catalytic activity of molybdenum disulfide (MoS_2_) by identifying and exposing active sites^6^,^16^, as well as enhancing electron conduction through nanostructuring^17^, shape control^6^,^18^, phase engineering^19^,^20^, doping^21^,^22^, intercalation^23^ and hybridization^17^,^24^,^25^. Recently a ternary cobalt phosphosulfide pyrite-type structure (CoPS) has been reported with superior catalytic activity for HER^26^. Cobalt disulfide (CoS_2_) is another material of interest^27^,^28^. However, the existing HER catalyst materials based on non-noble metal elements are still less satisfactory in terms of both activity and stability, which calls for further material structure innovation to realize efficient and cost effective HER catalysis.

Here we report a design and synthesis of a highly active and stable HER electrocatalyst material consisting of pyrite-structured cobalt phosphosulfide (CoS|P) nanoparticles anchored on carbon nanotubes (CNTs). The material architecture is built by a three-step chemical synthesis: Strong interactions with CNTs and particle size control are first established by the selective growth of cobalt(II,III) oxide (Co_3_O_4_) nanoparticles on CNTs; High catalytic activity for HER is then rendered by conversion of Co_3_O_4_ to CoS_2_ nanoparticles; Good chemical stability and catalytic durability are lastly obtained from substituting some of the sulfur with phosphorus. The unique material structure directly enables some of the highest HER catalytic performance among all Co-based catalyst materials. In 0.5 M H_2_SO_4_, the CoS|P/CNT hybrid exhibits a negligible onset overpotential and a Tafel slop of 55 mV per decade with an exchange current density of 1.14 mA cm^−2^. At a mass loading of 1.6 mg cm^−2^, the hybrid material requires overpotentials of only 48 mV and 109 mV to reach stable catalytic current densities of 10 mA cm^−2^ and 100 mA cm^−2^ respectively, representing one of the few most active non-Pt catalysts for HER. Phosphorus substitution in the pyrite structure is a critical step that renders chemical stability and catalytic durability to the CoS|P/CNT hybrid material. Density functional theory (DFT) calculations confirm the structural stability of pyrite CoS|P and suggest stronger metal–ligand bonding as a contributor to the improved stability, which is supported by X-ray absorption spectroscopy data.

## Results

### Sequential synthesis of CoS|P/CNT

**Figure 1:**
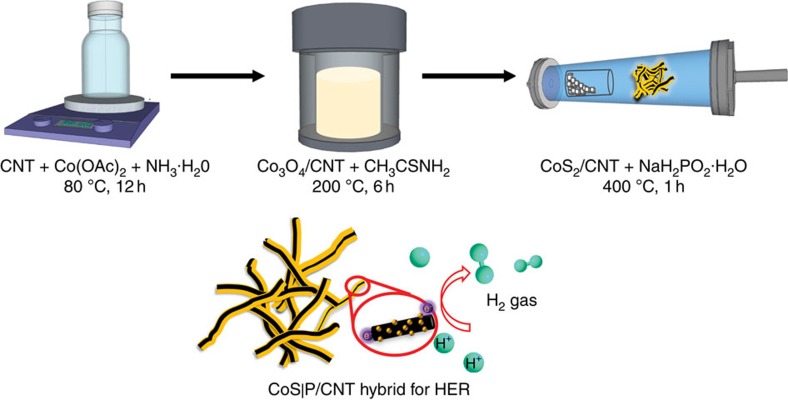
Schematic illustration of the sequential synthesis of the CoS|P/CNT hybrid material for HER catalysis. The CoS|P/CNT is synthesized through three steps including hydrolysis, hydrothermal sulfurization and solid/gas-phase phosphorization.

The synthetic strategy for the CoS|P/CNT hybrid material structure involves three steps, as illustrated in Fig. 1. In the first step, Co_3_O_4_ nanoparticles were directly and selectively grown onto mildly oxidized multi-wall CNTs (see Supplementary Methods for CNT oxidation) by a hydrolysis reaction of cobalt acetate at 80 °C. An ethanol/water mixed solvent was used to slow down the hydrolysis reaction, facilitate interactions between the Co^2+^ and the functional groups on the CNT surface, and ensure selective nucleation and growth of Co_3_O_4_ nanoparticles on CNTs. NH_3_·H_2_O was added into the reaction system to coordinate the Co^2+^ and thus further reduce the hydrolysis rate to limit the size of the resulting Co_3_O_4_ nanoparticles and optimize their distribution on the CNTs. Scanning electron microscopy (SEM), transmission electron microscopy (TEM) and X-ray diffraction (XRD) characterizations revealed the product structure as spinel structured Co_3_O_4_ (PDF#43-1003) nanoparticles with an average size of 5–10 nm anchored on CNTs (Supplementary Fig. 1).

The second step is a hydrothermal reaction at 200 °C to convert Co_3_O_4_ into CoS_2_. Thioacetamide (CH_3_CSNH_2_) was used as a slow-release S precursor, which reacted with water to gradually generate the H_2_S reactant and therefore allowed for facile chemical conversion from oxide to sulfide without damaging the material morphology and hybrid structure. SEM and TEM imaging showed nanoparticles with an average size of 10–20 nm attached to CNTs (Supplementary Fig. 2a,b). The nanoparticles were confirmed to be pyrite-structured CoS_2_ (PDF#41–1471) by XRD (Supplementary Fig. 2d). Lattice fringes of the CoS_2_ nanoparticles on CNTs were recorded with high-resolution TEM (Supplementary Fig. 2c). Energy dispersive spectroscopy (EDS) under scanning TEM (STEM) mode was used to map the distributions of elements of interest in the material structure. It is clear from the result that the nanoparticles consist of Co and S, and they are well-anchored on the CNT surface (Supplementary Fig. 3).

**Figure 2:**
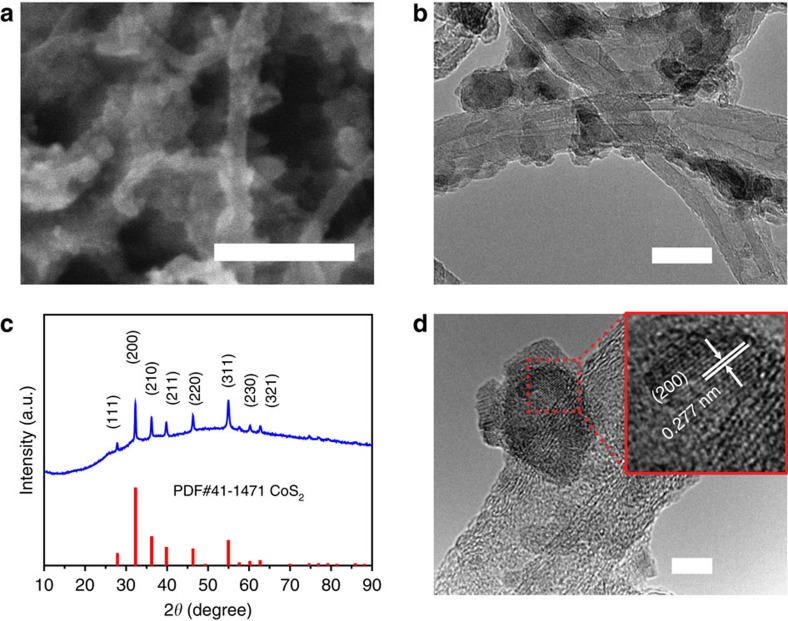
Structural characterizations of the CoS|P/CNT hybrid material. (**a**) SEM image of CoS|P/CNT; Scale bar, 200 nm. (**b**) Low-magnification TEM image of CoS|P/CNT showing nanoparticles attached to CNTs; Scale bar, 20 nm. (**c**) XRD pattern of CoS|P/CNT as compared with the pyrite-phase CoS_2_ standard (PDF#41–1471). (**d**) High-resolution TEM image showing the (200) lattice fringes of pyrite-phase CoS|P; Scale bar, 5 nm.

The third step features a solid/gas-phase reaction at 400 °C to introduce P into the CoS_2_ structure. NaH_2_PO_2_·H_2_O was used as a precursor to generate the PH_3_ reactant via thermal decomposition. The PH_3_ then reacted with the CoS_2_ nanoparticles on CNTs to form the final CoS|P/CNT hybrid material. SEM and TEM characterizations confirmed the microstructure of nanoparticles with an average size of 10–20 nm on CNTs (Fig. 2a,b), suggesting that the substitution process had negligible influence on the material morphology and nanoparticle size. XRD measurement of the material generated a diffraction pattern characteristic of a pyrite structure with almost identical lattice parameters as CoS_2_ (Fig. 2c), indicating that the substitution process happened in the form of P replacing S, which did not alter the crystal structure or lattice parameters due to the very similar atomic sizes of S and P. The lattice fringes of the CoS|P nanoparticles were imaged by high-resolution TEM (Fig. 2d). The interplanar spacing of 0.277 nm corresponding to the (200) crystallographic planes of the pyrite structure is in consistency with the XRD result. The high-resolution TEM images also excluded the existence of core-shell structured nanoparticles.

### Structural and chemical analysis

**Figure 3:**
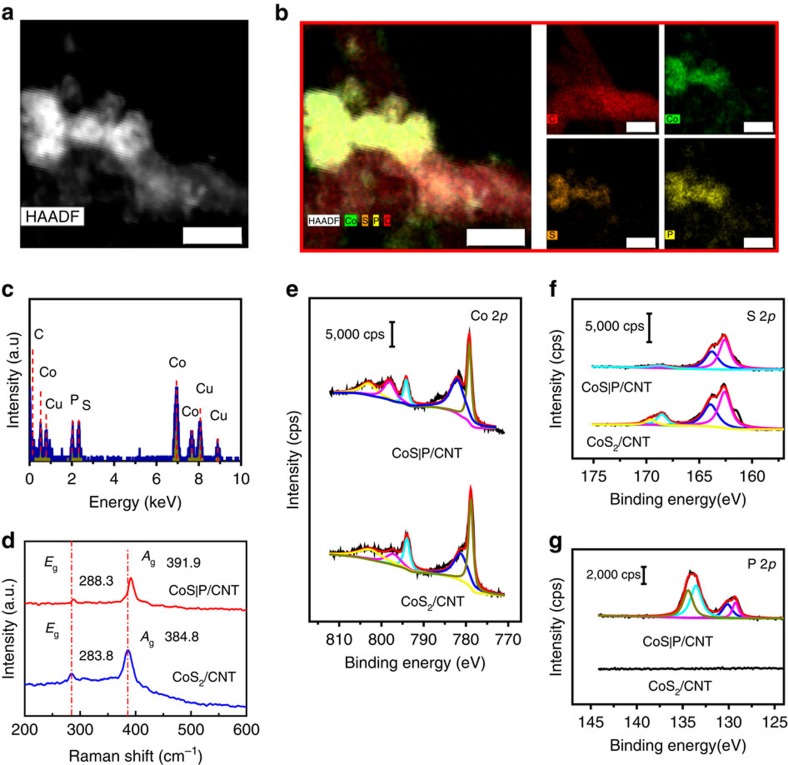
Composition and chemical analysis for the CoS|P/CNT hybrid material. (**a**) STEM image recorded by a high-angle annular dark field (HAADF) detector showing CoS|P nanoparticles attached on CNTs. Scale bar, 20 nm. (**b**) STEM-EDS mapping of CoS|P/CNT catalyst showing the distributions of Co (green), P (yellow) and S (orange) within the nanoparticles closely attached to C (red). Scale bar, 20 nm. (**c**) EDS spectrum of CoS|P/CNT. (**d**) Raman spectra of CoS_2_/CNT and CoS|P/CNT. (**e**–**g**) Co 2*p*, S 2*p* and P 2*p* core level XPS spectra of CoS|P/CNT and CoS_2_/CNT.

To further understand the structure of the CoS|P/CNT hybrid material, STEM-EDS characterization was performed to gain elemental composition and distribution information. The EDS maps of Co, S and P overlap quite well (Fig. 3a,b), suggesting P has been uniformly doped into the crystal structure of nanoparticles. It is also evident from the EDS mapping that the CoS|P nanoparticles are closely anchored onto the CNT surfaces. The average atomic ratio of P/S in the nanoparticles was calculated to be ∼1.0 from the EDS spectrum (Fig. 3c and Supplementary Fig. 4), giving a ∼50% substitution of the S sites. Raman spectroscopy was used to probe chemical bonding information in the CoS|P nanoparticles. For the CoS_2_/CNT, two peaks were observed at 284 and 385 cm^−1^ (Fig. 3d, blue trace). These Raman peaks could be attributed to the characteristic *E*_g_ and *A*_g_ vibrational modes corresponding to the in-phase stretching and pure libration of the S–S dumbbells in the pyrite structure^29^,^30^,^31^. For the CoS|P/CNT, the peaks slightly shifted to higher wavenumber (Fig. 3d, red trace) as a result of partial phosphorus substitution for sulfur and possible formation of P–S dumbbells. The characteristic Raman pattern also excludes formation of CoP^32^.

X-ray photoelectron spectroscopy (XPS) was employed to investigate the surface composition and oxidation states of the catalysts. For the CoS_2_/CNT, Co 2*p*_3/2_ and 2*p*_1/2_ core level peaks were observed at binding energies of 778.8 eV and 794.0 eV, respectively, together with satellite features (Fig. 3e), which match literature results on CoS_2_ (ref. 28). The Co 2*p* core level spectrum of the CoS|P/CNT hybrid is very similar to that of the CoS_2_/CNT, with binding energies of Co 2*p*_3/2_ and 2*p*_1/2_ peaks at 779.2 eV and 794.1 eV, respectively (Fig. 3e). The negligible change in the Co 2*p* spectrum verifies that the oxidation state of Co is not affected by P substitution. The S 2*p* core level spectrum of the CoS_2_/CNT shows lower binding energy components at 162.6 eV and 163.9 eV (S 2*p*_3/2_ and 2*p*_1/2_) attributed to sulfide species as well as higher binding energy components at 168.5 eV and 169.6 eV (S 2*p*_3/2_ and 2*p*_1/2_), respectively, characteristic of sulfate species (Fig. 3f). Existence of the sulfate components suggests that the CoS_2_/CNT catalyst is slightly oxidized on surface. Interestingly, no sulfate features were found in the S 2*p* core level spectrum of the CoS|P/CNT (Fig. 3f), indicating P doping could prevent CoS_2_ from oxidation under ambient conditions. The P 2*p* core level spectrum of the CoS|P/CNT displays two peak regions (Fig. 3g), with one centred at the binding energy of 129.3 and 130.1 eV (P 2*p*_3/2_ and 2*p*_1/2_), which can be assigned to phosphorus anions, and the other at 133.6 and 134.4 eV (unresolved doublet) characteristic of phosphate-like P. The existence of the high oxidation state P could be ascribed to surface oxidation under ambient conditions as often observed for metal phosphide materials^33^,^34^,^35^. Furthermore, a surface P/S ratio of ∼1.0 was derived from the XPS results, close to the bulk P/S ratio measured by EDS. This again corroborates that P is uniformly distributed within the nanoparticles rather than forming a shell-like structure on the original CoS_2_ nanoparticles.

### Electrocatalytic hydrogen evolution

**Figure 4:**
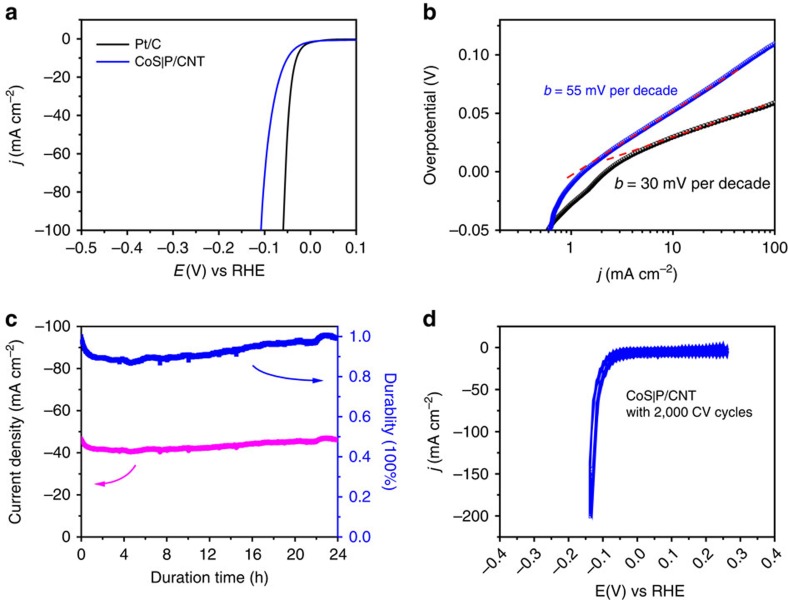
Electrocatalytic hydrogen evolution over the CoS|P/CNT catalyst. (**a**) Polarization curves for HER on the CoS|P/CNT hybrid and a commercial Pt/C catalyst at 5 mV s^−1^. The catalyst mass loading was 0.4 mg cm^−2^ for Pt/C catalyst. (**b**) Tafel plots for the CoS|P/CNT and Pt/C catalysts derived from the polarization curves in **a**. (**c**) Chronoamperometric response (*j*∼*t* curve) recorded on the CoS|P/CNT electrode at a constant overpotential of 95 mV with iR compensation. (**d**) CV test between 0.25 and −0.12 V versus RHE at a scan rate of 100 mV s^−1^ for 2,000 cycles. The catalyst mass loading of CoS|P/CNT was 1.6 mg cm^−2^ unless otherwise noted.

HER electrocatalytic activity of the CoS|P/CNT hybrid material was assessed in 0.5 M H_2_SO_4_ aqueous solution. Figure 4a shows the polarization curve of the CoS|P/CNT hybrid material as compared with a benchmark Pt/C catalyst at a scan rate of 5 mV s^−1^. With a mass loading of 1.6 mg cm^−2^, the CoS|P/CNT electrode showed a negligible onset overpotential versus the reversible hydrogen electrode (RHE). The catalytic current density increased rapidly with further cathodic polarization to 10 mA cm^−2^, 20 mA cm^−2^ and 100 mA cm^−2^ at overpotentials of 48 mV, 65 mV and 109 mV, respectively (Fig. 4a). An exchange current density of 1.14 mA cm^−2^ and a Tafel slope of 55 mV per decade were derived from the polarization curve (Fig. 4b), suggesting a different HER mechanism from Pt which showed a 30 mV per decade Tafel slope indicative of a Volmer–Tafel mechanism^36^. Such performance represents arguably higher HER catalytic activity than any other Co-based catalyst materials reported in the literature, placing our material at the top of all existing noble-metal-free HER catalyst materials working in acidic media (Supplementary Table 1). Our catalyst also showed high durability for HER catalysis. The initial current density (∼45 mA cm^−2^) maintained after 24 h of continuous hydrogen production (Fig. 4c). Stability for 100 h of HER catalysis was also confirmed (Supplementary Fig. 5). We also performed 2,000 cycles of cyclic voltammetry between 0.25 and −0.12 V versus RHE on the CoS|P/CNT catalyst. The polarization curves showed negligible shift during the test (Fig. 4d). Chronoamperometry in combination with gas chromatography and mass spectrometry (MS) revealed Faradic efficiency of ∼100% for H_2_ (Supplementary Fig. 6).

## Discussion

The excellent HER catalytic activity and durability of the CoS|P/CNT hybrid material is a direct outcome of its unique material structure imparted by the three designed chemical reaction steps. The first step of reaction grows Co_3_O_4_ nanoparticles on CNTs, which builds the strong electrical and chemical coupling between the nanoparticles and CNTs^37^,^38^. Consequently, nanoparticles are anchored on the highly conductive CNT network, which can rapidly transport electrons from external circuit to nanoparticle/electrolyte interface for hydrogen evolution. This step also brings size control of the nanoparticles, which is essential for increasing the electrochemically active surface area and reducing the electron diffusion length within each nanoparticle.

The second step of reaction converts the Co_3_O_4_ nanoparticles to CoS_2_ nanoparticles, through which highly active sites for HER catalysis are created. The CoS_2_/CNT material is already as active as the final CoS|P/CNT. At a mass loading of 0.8 mg cm^−2^, HER current density of 10 mA cm^−2^ was reached at an overpotential of 61 mV for the CoS_2_/CNT as compared with 64 mV for the CoS|P/CNT under the same condition (Supplementary Fig. 8a). This already represents one of the most active cobalt chalcogenide HER catalyst materials^10^,^28^,^39^,^40^,^41^,^42^. The sequential synthetic method, namely oxide growth followed by conversion to sulfide, is responsible for the superior catalytic activity, as corroborated by our control experiment (Supplementary Fig. 7).

**Figure 5:**
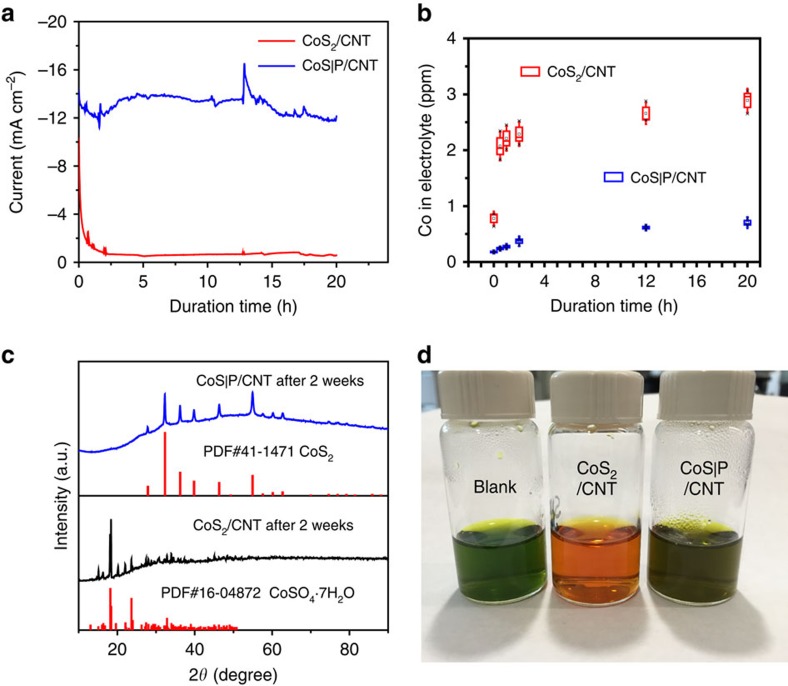
Comparison of chemical stability and catalytic durability between CoS_2_/CNT and CoS|P/CNT. (**a**) Typical chronoamperometric responses (*j*∼*t* curves) of the CoS_2_/CNT and CoS|P/CNT catalysts driving hydrogen evolution at the overpotential of 77 mV without iR compensation for 20 h in 0.5 M H_2_SO_4_ solution. About 0.4 mg of each catalyst was loaded on a carbon fibre paper with 0.5 cm^2^ of active area. The sharp current fluctuations were caused by the sampling of electrolyte during the electrolysis process. (**b**) Box plots (median and quartiles) representing the concentrations of Co dissolved in 20 ml of electrolyte as the HER catalysis proceeds. The vertical whiskers represent the s.d. The statistics are derived from at least three independent measurements. (**c**) XRD patterns of CoS_2_/CNT and CoS|P/CNT after 2 weeks of storage in ambient conditions. (**d**) Colorimetric comparison of the CoS_2_/CNT and CoS|P/CNT hybrids soaked in 0.5 M H_2_SO_4_ solution for 2 h; Nitrite R salt was used as the colour indicator.

In spite of the excellent activity as a result of size control and CNT hybridization, the intrinsic instability of CoS_2_ in strong acid is exacerbated. The CoS_2_/CNT material is extremely unstable during HER catalysis in 0.5 M H_2_SO_4_. Under constant potential operation, the current density decreased drastically by 70% in less than 30 min (Fig. 5a). Such a dramatic deterioration was accompanied by substantial dissolution of the CoS_2_ active phase into the electrolyte. A Co concentration of ∼2.1 p.p.m. in the electrolyte was measured by inductively coupled plasma MS (ICP-MS) after 30 min of HER catalysis (Fig. 5b), corresponding to about 17.5% of the CoS_2_ having been dissolved. The concentration of dissolved Co gradually increased to ∼2.9 p.p.m. over 20 h of HER operation. The CoS_2_/CNT material is also sensitive to air and moisture. After being stored in ambient condition for 2 weeks, the CoS_2_ nanoparticles were completely oxidized to CoSO_4_·7H_2_O (PDF#16-04872), which is soluble in water and HER inactive (Fig. 5c).

The third step of reaction (P substitution) is critical to the chemical stability and catalytic durability of the final material (Fig. 5 and Supplementary Fig. 8). No phase change was detected by XRD for the CoS|P/CNTs material after being stored in ambient condition for 2 weeks (Fig. 5c), suggesting significantly improved chemical stability to oxygen and moisture. This is consistent with our XPS results that P substitution could mitigate sulfate formation on CoS_2_ surface. The CoS|P/CNTs catalyst was able to sustain a current density of ∼10 mA cm^−2^ during 20 h of continuous HER operation (Fig. 5a), substantially more stable than the CoS_2_/CNT catalyst under working conditions. In consistency, the amount of Co leaching into the electrolyte was much lower than that for the CoS_2_/CNT catalyst. The Co concentration in electrolyte gradually reached ∼0.6 p.p.m. within 12 h and did not increase significantly in the following 8 h (Fig. 5b). To visually demonstrate the stability difference between the CoS_2_/CNT and CoS|P/CNT in the electrolyte, we conducted a colorimetric comparison (Supplementary Methods) after the CoS_2_/CNT and CoS|P/CNT hybrids were soaked in 0.5 M H_2_SO_4_ for 2 h. The Nitrite R salt is widely used as a colour indicator to detect cobalt in solution as it can form a red coloured complex with Co^2+^ ions. The solution in which the CoS_2_/CNT was soaked exhibited an orange colour (Fig. 5d), clearly showing a considerable amount of Co dissolved into the acid. In contrast, the solution in which the CoS|P/CNT was soaked remained almost the same colour as the blank control, confirming greatly improved stability of the CoS|P/CNT catalyst against acid corrosion. Our control experiment clearly verified that it is indeed the P substitution rather than the high-temperature annealing process that renders the excellent catalytic durability (Supplementary Fig. 9).

It is worth emphasizing here that the P substitution step is only for improving material stability and catalytic durability, but not for enhancing catalytic activity. This is different from the recent study where the CoPS is much more active than the CoS_2_ with similar morphology^26^. It is also noted that our CoS|P is likely in a different structure than the recently reported CoPS. The CoPS is a distinct ternary pyrite-type phase featuring P–S bonds without any S–S bonds as in the CoS_2_ pyrite structure. The corresponding XRD and Raman peaks significantly shifted as a result of the shrunk lattice parameters. In contrast, our CoS|P is more likely to be a CoS_2_ pyrite structure (no lattice parameter shrinking) with some of the S atoms randomly substituted by P atoms. The structural difference is a direct outcome of the different synthetic methods adopted. The CoPS is prepared by converting Co(OH)(CO_3_)_0.5_. XH_2_O with pre-formed P_*x*_S_*y*_, therefore the P–S bonding is exclusive and CoPS is the only available composition. In this study, CoS|P is derived from CoS_2_ so that the P/S ratio in the CoS_2−*x*_P_*x*_/CNT materials can be readily tuned between 0 and 1 while keeping the mother structure and lattice parameters unchanged (Supplementary Fig. 10). As we gradually increase the substitution level of P, the influence on HER activity is negligible but the catalytic durability improves drastically (Supplementary Fig. 11).

**Figure 6:**
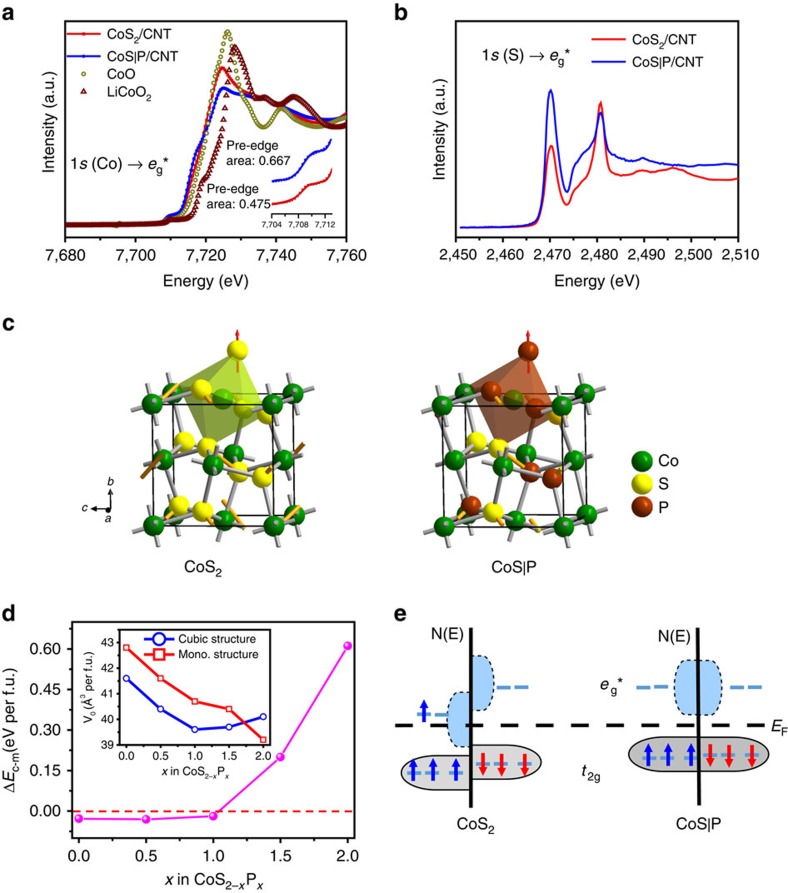
XANES spectra of CoS|P/CNT and structural stability discussion based on first-principles calculations. (**a**) Cobalt K-edge XANES spectra of CoS_2_/CNT and CoS|P/CNT compared with Co^II^ (CoO) and Co^III^ (LiCoO_2_) standards. Pre-edge features correspond to transition from 1*s* (Co) orbital to *e*_g_* anti-bonding state, which is metal 3*d* and ligand 3*p* hybrid orbitals. The pre-edge features were fitted by two pseudo-Voigt functions with the results shown in the inset graph. (**b**) Sulfur K-edge XANES spectra of CoS_2_/CNT and CoS|P/CNT. (**c**) Structure illustration of pyrite-phase CoS_2_ and CoS|P (CoS_2−*x*_P_*x*_, *x*=1), each with a representative coordination polyhedron. (**d**) The energy difference per formula unit (f.u.) between the cubic and monoclinic phases as a function of the P substitution extent obtained from DFT calculations. The inset shows the equilibrium volume per f.u. in the cubic and monoclinic phases as a function of the P substitution extent. (**e**) Conceptual energy level diagrams of the frontier molecular orbitals for pyrite-phase CoS_2_ and CoS|P derived from the calculated electronic structures (Supplementary Fig. 12). CoS_2_ is magnetic at room temperature. As a result spin-up and spin-down electrons have different energy levels. CoS|P (CoS_2−*x*_P_*x*_, *x*=1) is non-magnetic.

To probe the molecular origins of the structural stability of CoS|P/CNT, we performed X-ray absorption near edge structure (XANES) spectroscopy measurements. The absorption edges of the Co K-edge spectra of CoS_2_/CNT and CoS|P/CNT lie close to each other (Fig. 6a), suggesting similar oxidation states of Co in both materials, which match the XPS results. The relatively small pre-edge peaks of both spectra suggest that the Co ions invariably reside in octahedral coordination environment (Fig. 6c). It is noted that the CoS|P/CNT spectrum shows a shoulder peak at ∼7,717 eV. Similar features have been observed in other systems and are attributed to the covalency effect^43^. This correlates well with the pre-edge absorption intensity difference between the CoS|P/CNT and CoS_2_/CNT (inset of Fig. 6a). For both CoS|P and CoS_2_, the lowest unoccupied molecular orbitals are the anti-bonding *e*_g_* orbitals^44^. These orbitals are due to hybridization between transition metal 3*d* and ligand 3*p* orbitals and are thus very sensitive to covalency. The stronger pre-edge adsorption of CoS|P/CNT compared with that of CoS_2_/CNT suggests more p features in the Co 3*d* state and therefore stronger covalency for the CoS|P compound. Stronger covalency between the transition metal and ligands in CoS|P/CNT is also supported by the S K-edge XANES spectra. As shown in Fig. 6b, the CoS|P/CNT spectrum exhibits a stronger peak at ∼2,469 eV than the CoS_2_/CNT, corresponding to higher probability of 1*s* to *e*_g_* transition as a result of stronger covalency of the metal–ligand bonds in CoS|P^45^.

To further rationalize the experimental findings, we performed first-principles DFT calculations for CoS_2−*x*_P_*x*_ (*x*=0, 0.5, 1.0, 1.5 and 2) with both the cubic (pyrite) and monoclinic structures. The results for the relative stability of the two phases are shown in Fig. 6d. We found that CoS_2_ is more stable in the cubic structure than in the monoclinic structure, but only with a marginal energy difference of about 0.03 eV per formula unit (f.u.). In contrast, CoP_2_ is substantially more stable in the monoclinic structure by about 0.6 eV per f.u. When S is substituted by P, the phosphosulfides would be more stable in the monoclinic structure within a large range of *x* if we assume a linear dependence of the relative stability of the two phases on the composition. However, first-principles calculations clearly suggest that CoS_2−*x*_P_*x*_ is more stable in the cubic structure for *x*≤1.0, which agrees well with our experimental results. The remarkable stability of the cubic structure can be attributed to the fact that cubic-phase CoS_2_ and CoP_2_ have very similar optimized equilibrium volume (*V*_0_) (41.6 versus 40.1 Å^3^ per f.u.). In comparison, the *V*_0_ difference between monoclinic-phase CoS_2_ and CoP_2_ is much larger (42.8 versus 39.2 Å^3^ per f.u.). Substitution of S by P would induce much less strain in the cubic than in the monoclinic structure. Therefore, the pyrite-structured CoS_2−*x*_P_*x*_ is stable within a quite wide range of P doping levels (*x*≤1.0).

First-principles calculations can also provide insight into why incorporation of P improves the chemical stability of pyrite-structured CoS_2_. From projected density of states analysis (Supplementary Fig. 12), we see that P substitution significantly influences the nature of chemical bonding between Co and S/P. In the pyrite structure, each Co atom is coordinated in an octahedral ligand field (Fig. 6c), and therefore the 3*d* orbitals are split into *t*_2g_ and *e*_g_* manifolds that are of non-bonding and anti-bonding characteristics, respectively. As qualitatively demonstrated in Fig. 6e, the highest occupied states in CoS_2_ are of anti-bonding nature, which is the origin of the instability. When half of the S atoms are replaced by P, which has fewer valence electrons, the anti-bonding *e*_g_* orbitals are depleted, which strengthens the chemical bonding between Co and ligands and thus enhances the chemical stability of the material. This is in good agreement with our XANES results. In conclusion, we demonstrate a novel structure design and synthesis of a highly active and stable HER catalyst material based on pyrite-structured CoS|P. The sequential synthetic strategy we adopt imparts electrical conductivity, catalytic activity and stability to the material. The CoS|P/CNT catalyst exhibits arguably the highest catalytic activity among all non-noble metal based catalysts. P substitution is critical to chemical stability and catalytic durability of the material. The molecular origins are rationalized by spectroscopy characterization and computational modelling.

## Methods

### Material synthesis

The CoS|P/CNT hybrid was prepared through a three-step method. In the first step (synthesis of Co_3_O_4_/CNT), 4 mg of mildly oxidized CNTs (the CNTs were oxidized following a modified Hummers method as described in Supplementary Information) were dispersed in 14 ml of ethanol by sonication for 1 h. Then, 0.8 ml of 0.2 M cobalt acetate aqueous solution and 0.6 ml of NH_4_OH (30%) were added to the suspension. The hydrolysis reaction was kept at 80 °C in oil bath with stirring for 12 h. After that, the product was collected by centrifuge. The precipitate was then washed with ethanol and DI water. The resulting Co_3_O_4_/CNT was lyophilized. To prepare CoS_2_/CNT, 20 mg of Co_3_O_4_/CNT was dispersed in 20 ml of DI water by sonication for 40 min, followed by the addition of 0.75 ml of 1 M thioacetamide solution. After that, the reaction mixture was transferred to a 40 ml autoclave for hydrothermal reaction at 200 °C for 6 h. The resulting product was collected by centrifugation and repeatedly washed with DI water. The CoS_2_/CNT hybrid was then freeze-dried. In the third step, 5 mg of CoS_2_/CNT and 100 mg of NaH_2_PO_2_·H_2_O were placed at two separate positions in a ceramic crucible with the NaH_2_PO_2_·H_2_O at the upstream side. The samples were heated at 400 °C for 1 h with Ar gas flowing at 200 s.c.c.m. The final product contains about 60 wt% of CoS|P and 40 wt% of CNTs.

### Electrochemical measurements

To prepare catalyst ink, 1 mg of CoS|P/CNT was mixed with 190 μl of water, 50 μl of ethanol and 10 μl of 5 wt% Nafion solution by sonication for 1 h. Subsequently, 50–200 μl of the catalyst ink was drop-dried onto a carbon fibre paper (Spectracarb 2050 A from Fuel Cell Store) to cover an area of 0.5 cm^2^ (0.4–1.6 mg cm^−2^). The electrode was further heated at 90 °C in vacuum for 2 h. HER catalytic measurements were performed with a CHI 760D electrochemistry workstation (CH instruments, USA). A conventional three electrode cell configuration was employed. A saturated calomel electrode (SCE) was used as the reference electrode, and a graphite rod was used as the counter electrode. 0.5 M H_2_SO_4_ solution was used as electrolyte. Linear sweep voltammetry was recorded at a scan rate of 5 mV s^−1^. All the polarization curves were iR-corrected. The reference electrode was calibrated against the RHE as shown in Supplementary Fig. 13. All the potentials reported in our work were converted according to E (versus RHE)=E (versus SCE)+0.278 V.

### First-principles calculations

All DFT calculations were conducted by using the Vienna *ab initio* simulation package (VASP) suite that is based on the projector-augmented wave (PAW) approach and the plane wave basis set^46^. The structures of CoS_2−*x*_P_*x*_ (*x*=0, 0.5, 1.0, 1.5 and 2), including lattice constants and internal coordinates, in both the cubic (pyrite) and monoclinic phases, were optimized using the Perdew–Burke–Ernzerhof^47^ approximation for the exchange-correlation functional with a plane wave energy cutoff of 400 eV, which is about 1.5 times of the default cutoff value such that the Pulay stress problem can be avoided. To account for the possible strong correlation effects in the Co 3*d* electrons, the energy difference between the cubic and monoclinic phases was calculated using the PBE plus the Hubbard *U* correction (PBE+*U*) approach in the rotationally invariant scheme (LDAUTYPE=1)^48^ with *U*=4.5 eV and *J*=0.5 eV. We used the special quasi-random structure approach^49^ implemented in the ATAT code^50^ to model the random substitution of S by P in CoS_2−*x*_P_*x*_ using a supercell of 24 atoms.

## Additional information

**How to cite this article:** Liu, W. *et al.* A highly active and stable hydrogen evolution catalyst based on pyrite-structured cobalt phosphosulfide. *Nat. Commun.* 7:10771 doi: 10.1038/ncomms10771 (2016).

## Supplementary Material

Supplementary Information Supplementary Figures 1-13, Supplementary Table 1, Supplementary Methods and Supplementary References
