# Supplementary material for: A highly active and stable hydrogen evolution catalyst based on pyrite-structured cobalt phosphosulfide
Source: Nat Commun. 2016 Feb 19;7:10771. doi: 10.1038/ncomms10771 (PMC4762894; doi:10.1038/ncomms10771)
Supplement: Supplementary Information — Supplementary Figures 1-13, Supplementary Table 1, Supplementary Methods and Supplementary References [file ncomms10771-s1.pdf]

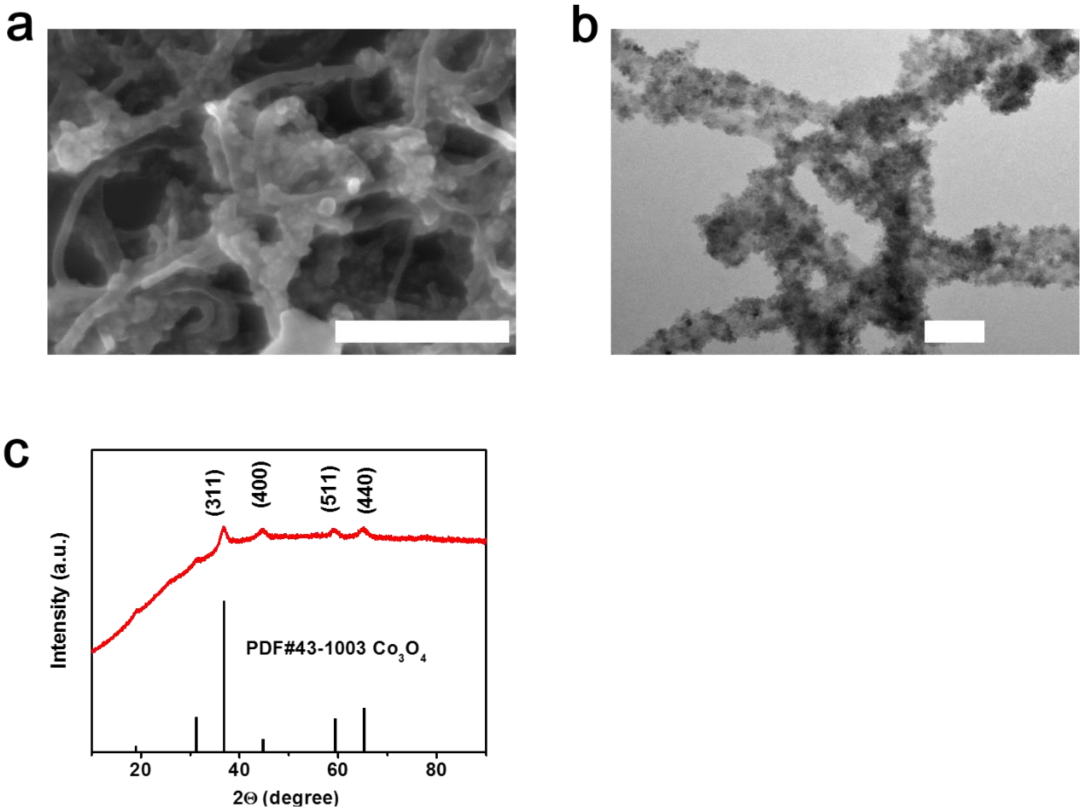

Supplementary Figure 1 | Morphology and crystal structure of Co_3O_4/CNT. (a) SEM image of Co_3O_4/CNT; Scale bar, 300 nm. **(b)** TEM image of Co_3O_4/CNT; Scale bar, 40 nm. **(c)** XRD patterns of Co_3O_4/CNT.

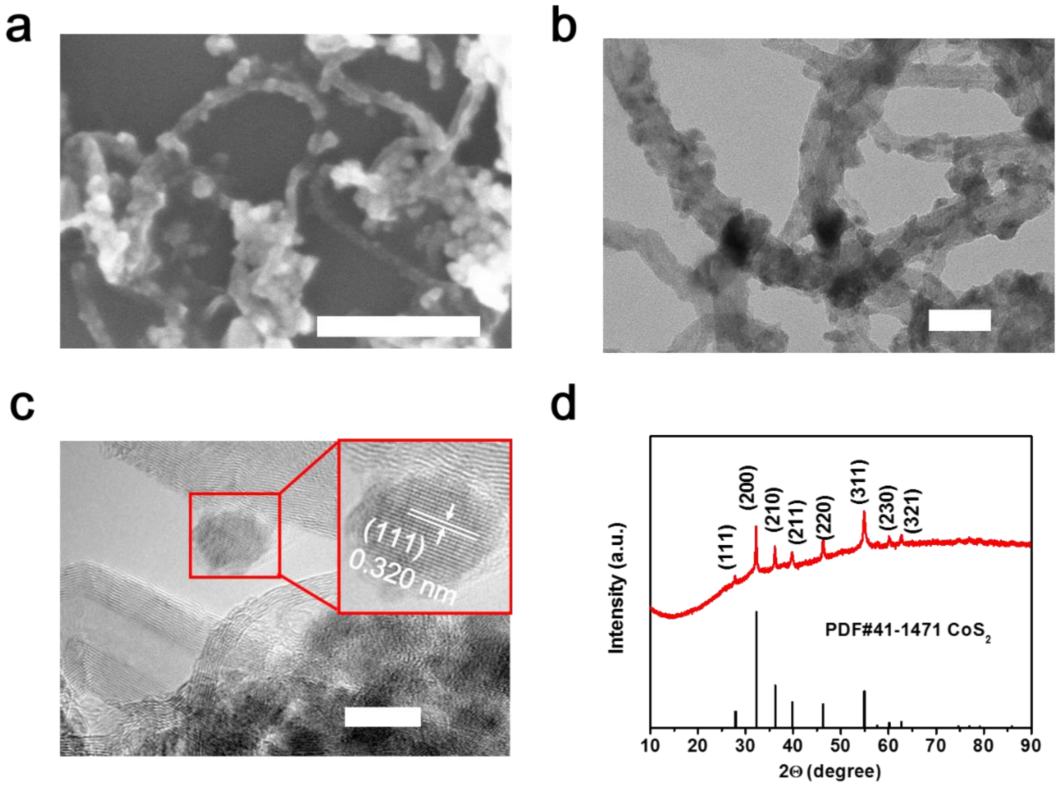

Supplementary Figure 2 | Morphology and crystal structure of CoS_2/CNT. (a) SEM image CoS_2/CNT; Scale bar, 300nm. **(b)** TEM image CoS_2/CNT; Scale bar, 40 nm. **(c)** High-resolution TEM image CoS_2/CNT; Scale bar, 20 nm. **(d)** XRD patterns of CoS_2/CNT.

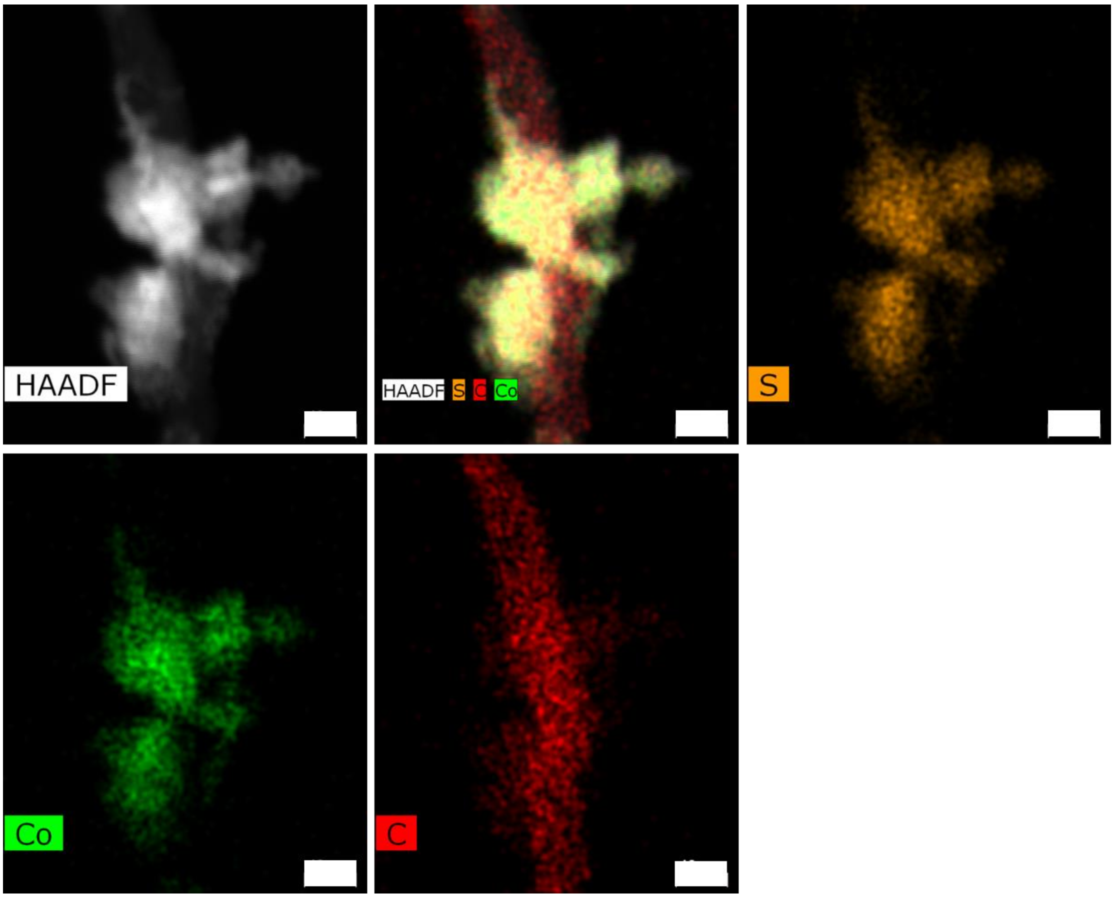

Supplementary Figure 3 | STEM-EDX elemental mapping of the CoS_2/CNT hybrid. STEM-HAADF image and corresponding EDX mapping of S (orange), Co (green), and C (red) element in the CoS_2/CNT hybrid. Scale bars, 10 nm.

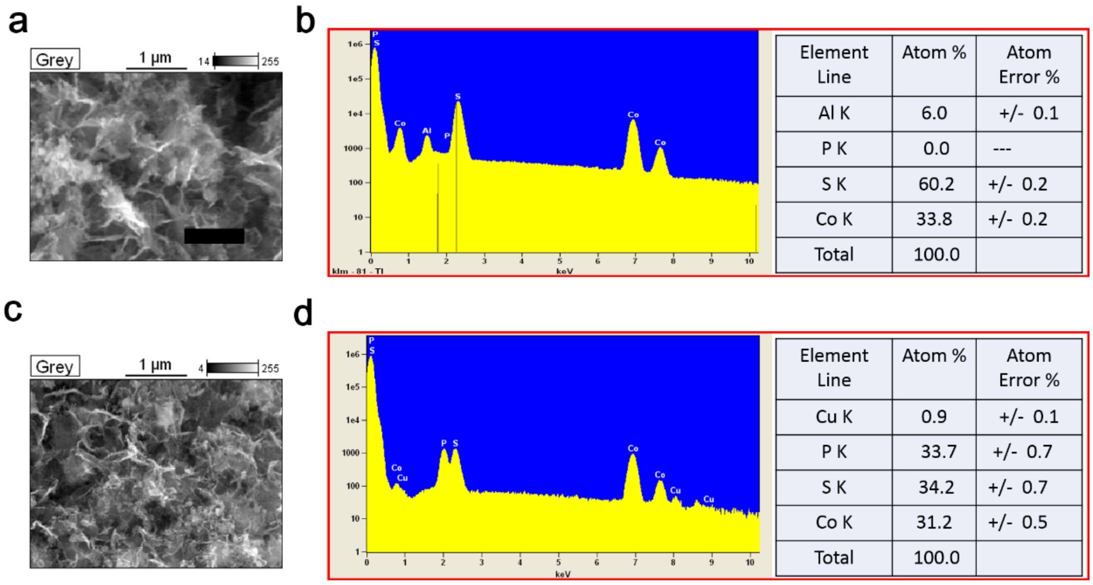

Element Line	Atom %	Atom Error %
Al K	6.0	+/- 0.1
P K	0.0	---
S K	60.2	+/- 0.2
Co K	33.8	+/- 0.2
Total	100.0	

Element Line	Atom %	Atom Error %
Cu K	0.9	+/- 0.1
P K	33.7	+/- 0.7
S K	34.2	+/- 0.7
Co K	31.2	+/- 0.5
Total	100.0	

Supplementary Figure 4 | SEM-EDS characterization of the CoS_2/CNT and CoS|P/CNT hybrids. (a, b) SEM image and corresponding EDS spectrum of CoS_2/CNT. (c, d) SEM image and corresponding EDS spectrum of CoS|P/CNT.

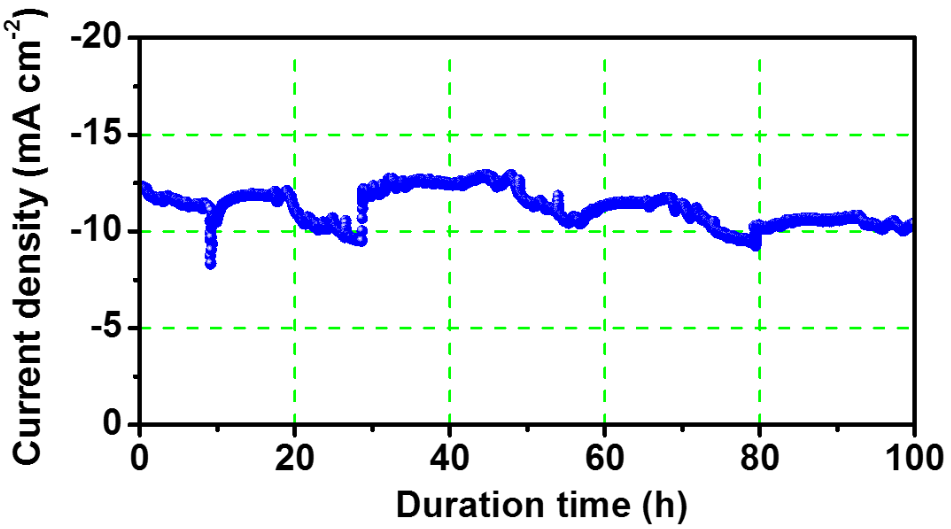

Supplementary Figure 5 | Long time catalytic stability of the CoS|P/CNT hybrid. Constant overpotential was employed to drive continuous hydrogen evolution reaction. After 100 hours of hydrogen production, the catalyst still maintained 84% of its initial catalytic current density (from 12.3 mA cm^{-2} to 10.4 mA cm^{-2}). The fluctuation of current density in the catalytic process may be caused by bubble formation and collapse.

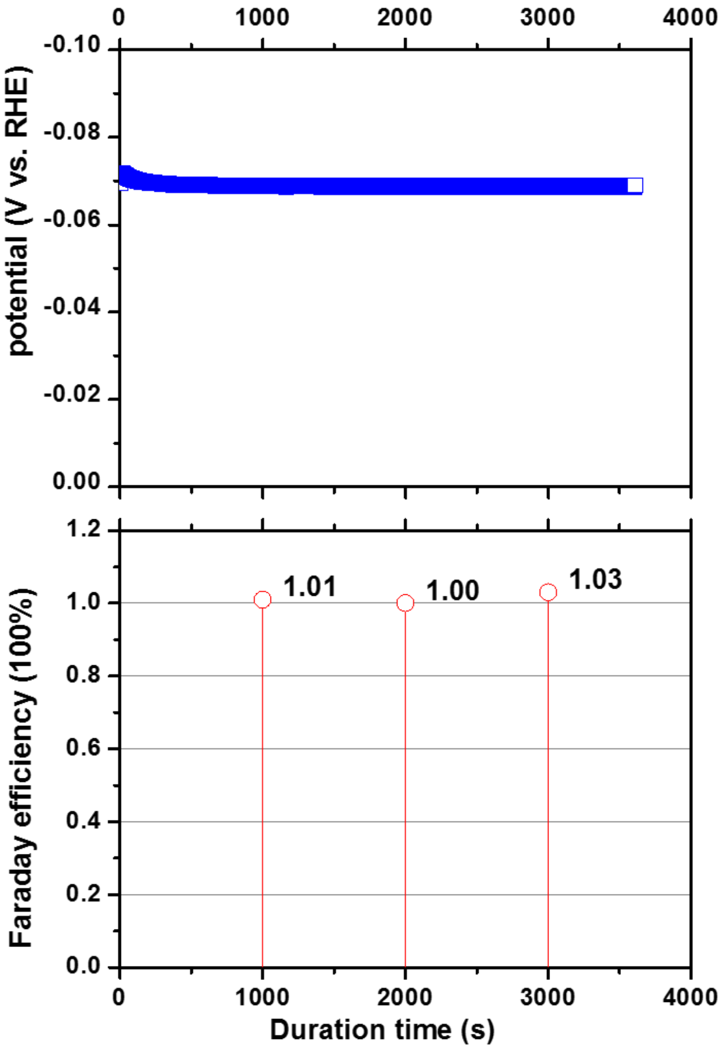

Supplementary Figure 6 | Faradaic efficiency for HER catalyzed by CoS|P/CNT. The actual amount of H_2 detected by GC-MS was compared with the current density (20 mA cm^{-2}) to derive the Faradaic efficiency.

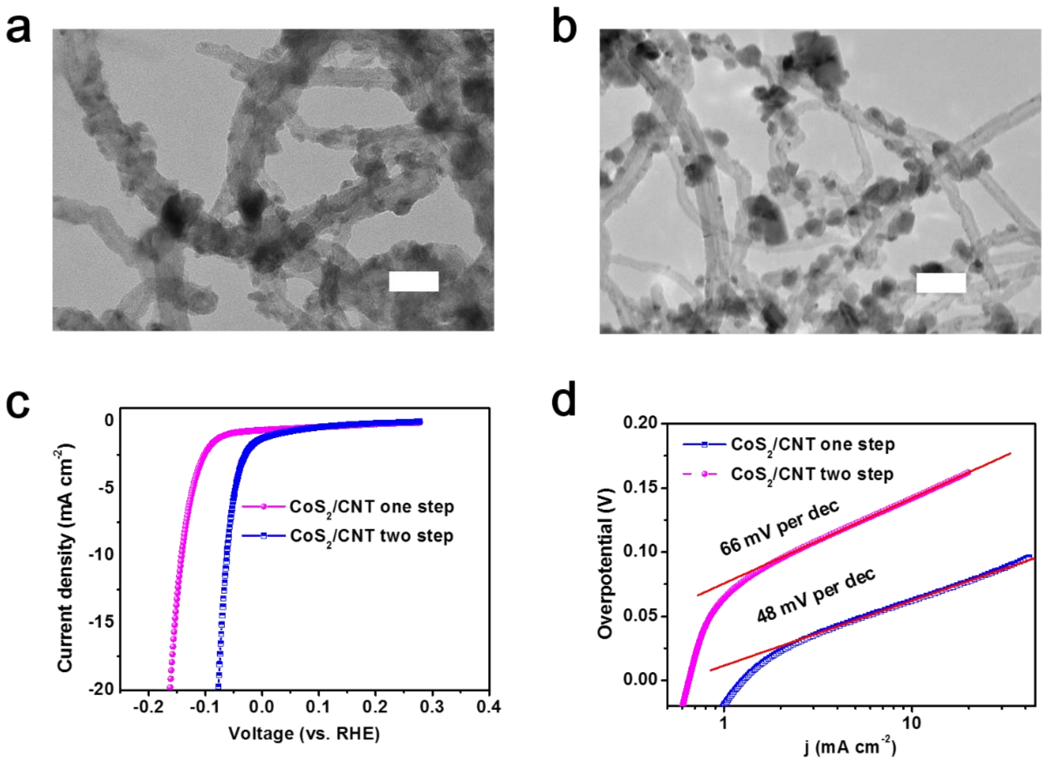

Supplementary Figure 7 | Comparison of CoS_2/CNT hybrids prepared using one-step and two-step methods. (a) TEM image of the CoS_2/CNT hybrid prepared with the two-step method. Scale bar, 40 nm. (**b)** TEM image of another CoS_2/CNT hybrid prepared using a one-step hydrothermal reaction; Scale bar, 40 nm. **(c)** The polarization curves of the CoS_2/CNT hybrids prepared with one-step and two-step methods. Catalyst mass loading was 0.8 mg cm^{-2}. **(d)** Corresponding Tafel slopes of the CoS_2/CNT hybrids derived from **(c)**.

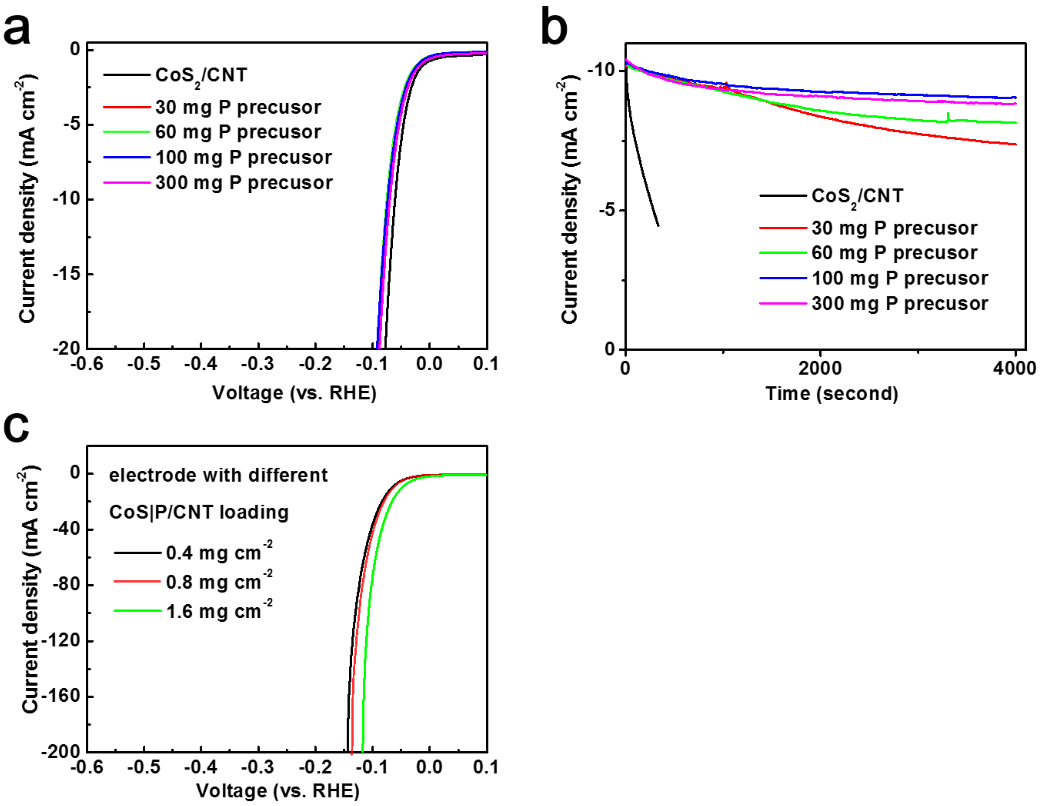

Supplementary Figure 8 | Catalytic activity and durability of CoS_2/CNT and $CoS_{2-x}P_x$/CNT hybrids with different P/S ratios and different catalyst loadings. (a) Polarization curves of $CoS_{2-x}P_x$/CNT hybrids prepared with different P dosages where 30, 60, 100 and 300 mg of $NaH_2PO_2{\cdot}H_2O$ salt were used for 5 mg of CoS_2/CNT; The CoS_2/CNT was used as a comparison. The catalyst mass loading was 0.8 mg cm^{-2}. **(b)** The durability (j ~ t curves) of $CoS_{2-x}P_x$/CNT hybrids with different P/S ratios. The catalyst mass loading was 0.8 mg cm^{-2}. The curves were recorded without iR compensation. **(c)** Polarization curves of CoS|P/CNT ($CoS_{2-x}P_x$/CNT with 100 mg P precursor) electrodes with different catalyst mass loadings.

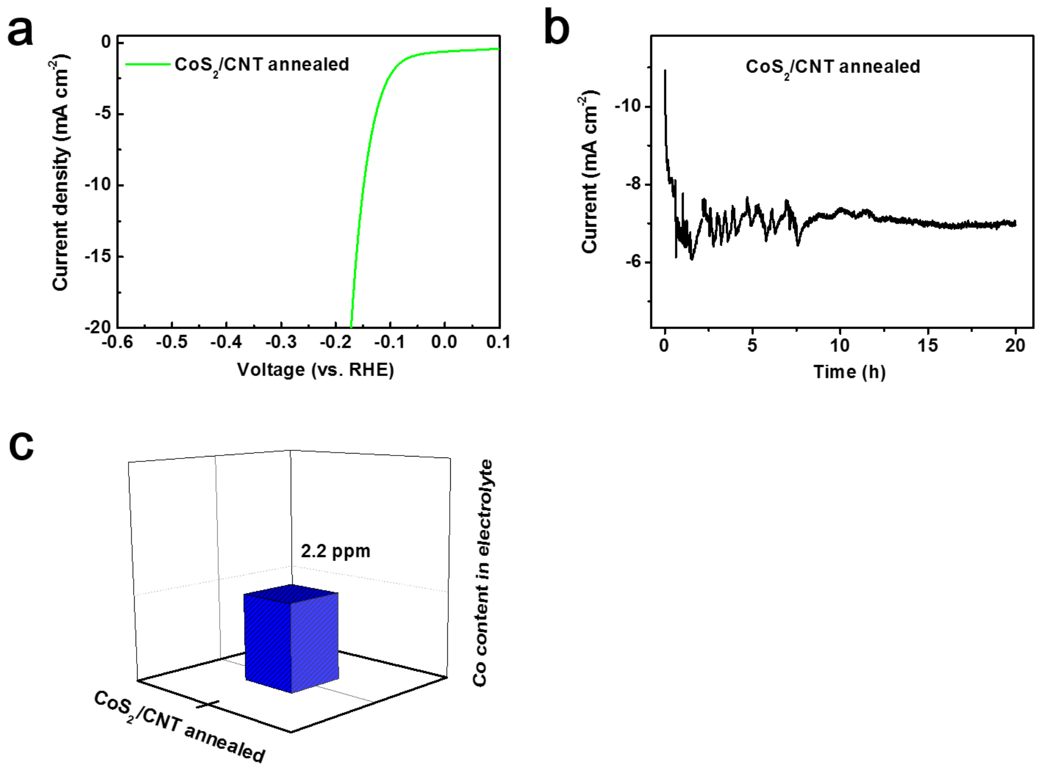

Supplementary Figure 9 | Catalytic activity and durability of the CoS_2/CNT hybrid annealed in Ar at 400 °C. (a) Polarization curve of CoS_2/CNT after annealing in Ar at 400 °C for 1 h, showing lower HER activity compared to the as-made CoS_2/CNT hybrid; the catalyst mass loading was 0.8 mg cm^{-2}. **(b)** Durability of the annealed CoS_2/CNT hybrid for HER in 0.5 M H_2SO_4 solution, showing that the stability of the annealed CoS_2/CNT is inferior to CoS|P/CNT; 0.4 mg of catalyst was loaded on a carbon fiber paper with 0.5 cm^2 of active area. The test was performed without iR compensation. **(c)** The concentration of Co dissolved in electrolyte for the annealed CoS_2/CNT hybrid after 20 h of HER electrolysis corresponding to the j ~ t curve in **(b)**.

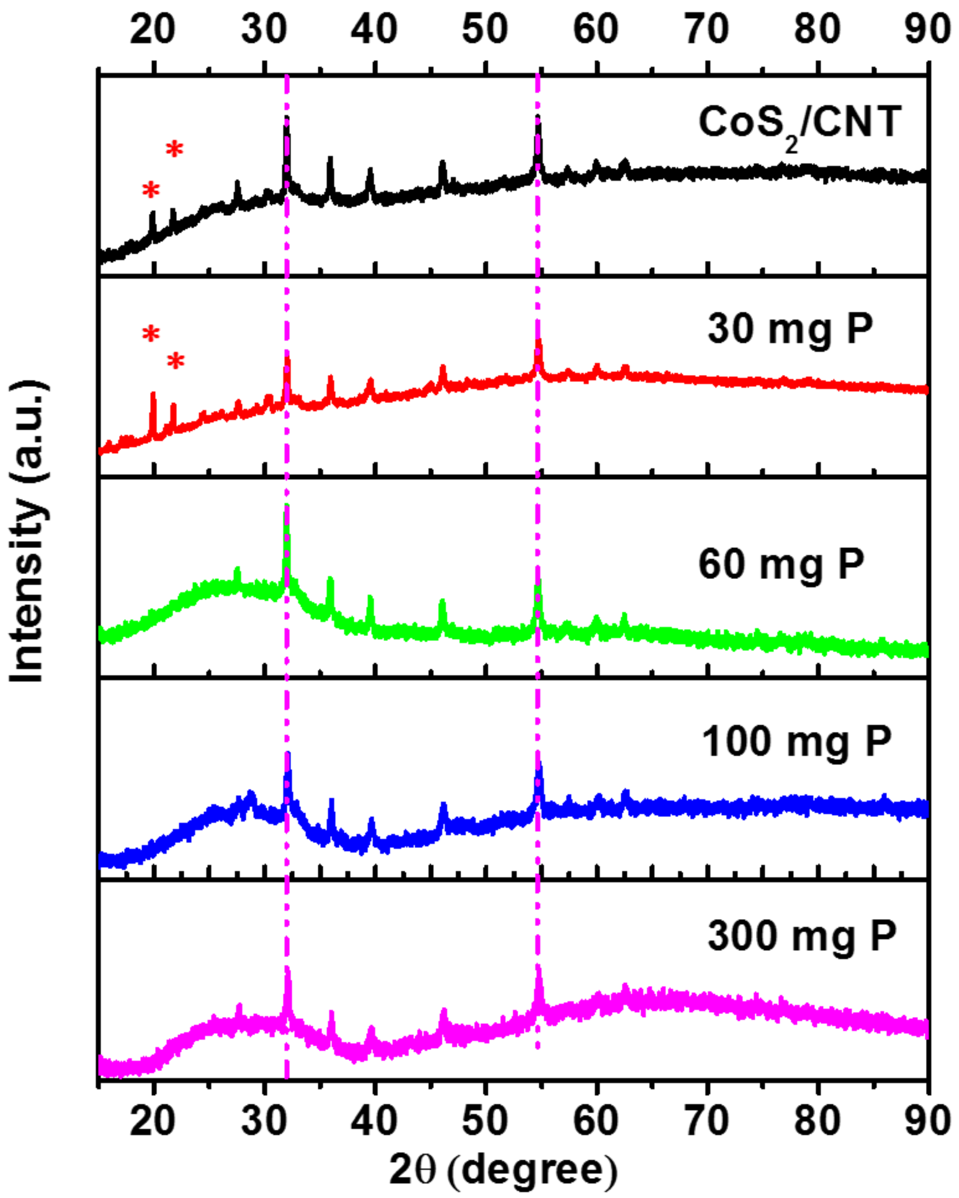

Supplementary Figure 10 | XRD patterns of the CoS_2/CNT and $CoS_{2-x}P_x$/CNT hybrids with different P/S ratios. In the study, the P/S ratio of the $CoS_{2-x}P_x$/CNT can be readily tuned between 0 and 1 by varying the dosages of $NaH_2PO_2·H_2O$ used. The diffraction patterns remain almost unchanged from the original CoS_2/CNT within the P/S range studied. The diffraction peaks marked with asterisk symbols in the XRD patterns of CoS_2/CNT and 30 mg P can be assigned to $CoSO_4·4H_2O$ (PDF#16-0488) which was formed from partially oxidation of pyrite CoS_2 in ambient condition. However, for the samples with higher P doping extents, no sulfate phase was detected. This confirms the role of P doping on the chemical stability of CoS|P.

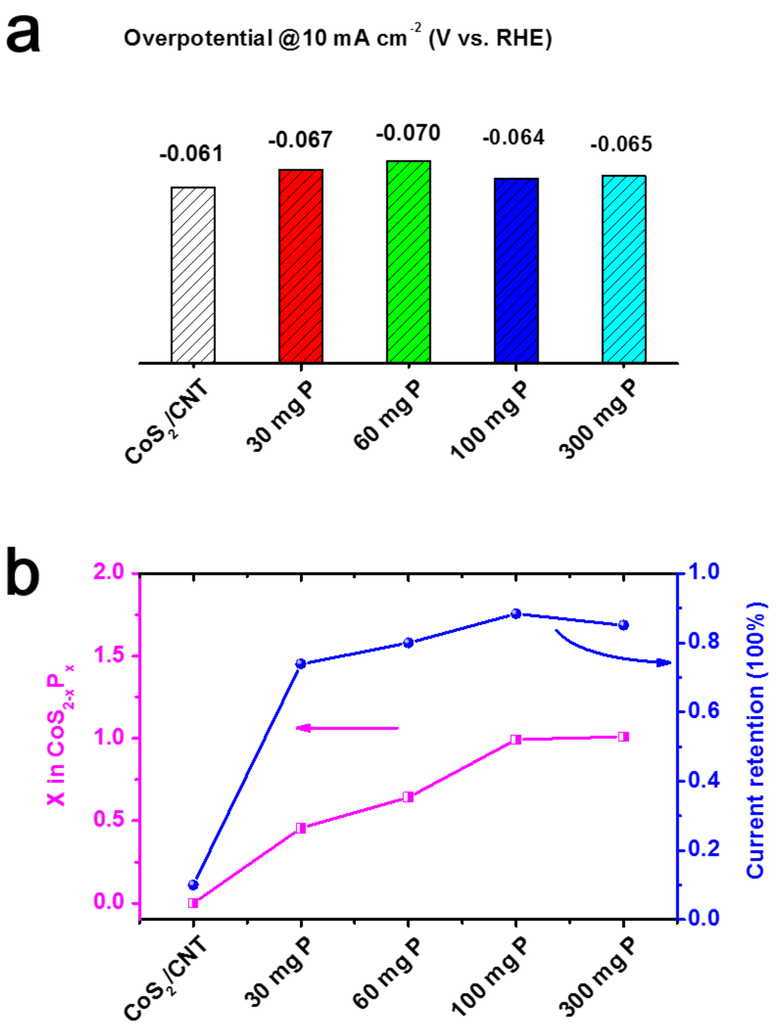

Supplementary Figure 11 | Catalytic activity and durability (derived from Supplementary Figure 8) of CoS_2/CNT and $CoS_{2-x}P_x$/CNT hybrids with different P/S ratios. (a) Potentials needed for the CoS_2/CNT and $CoS_{2-x}P_x$/CNT hybrids with different P doping levels to drive an HER current density of 10 mA cm^{-2}, showing that the catalytic activity is not affected by P substitution. **(b)** The correlation between catalytic durability and P content of the $CoS_{2-x}P_x$/CNT hybrids. After driving HER at ~10 mA cm^{-2} for 3600 seconds, the retention of catalytic current was compared among the catalysts with different P/S ratios. The P content in the $CoS_{2-x}P_x$/CNT hybrids increased with increasing P precursor dosage used in the synthesis until 50% of S was substituted with P (x=1.0 for $CoS_{2-x}P_x$). Further increase of the P dosage could not increase the P/S ratio of the $CoS_{2-x}P_x$/CNT. The catalytic stability of the $CoS_{2-x}P_x$/CNT was drastically enhanced by P doping.

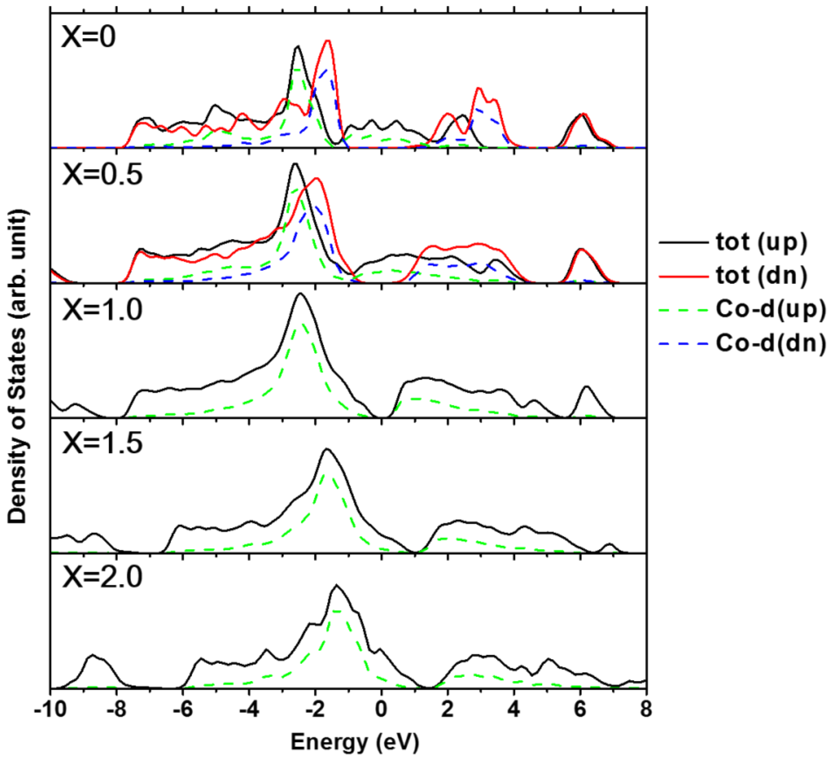

Supplementary Figure 12 | Spin-resolved projected density of states (PDOS) for $CoS_{2-x}P_x$ obtained from PBE+*U* calculations. Please note for x = 0 and 0.5, the ground states are magnetic, while for x = 1.0, 1.5 and 2 the ground states are non-magnetic. In the figure, tot (up), tot (dn), Co-d (up) and Co-d (dn) stand for total spin-up density of states, total spin-down density of states, Co 3d spin-up density of states and Co 3d spin-down density of states, respectively.

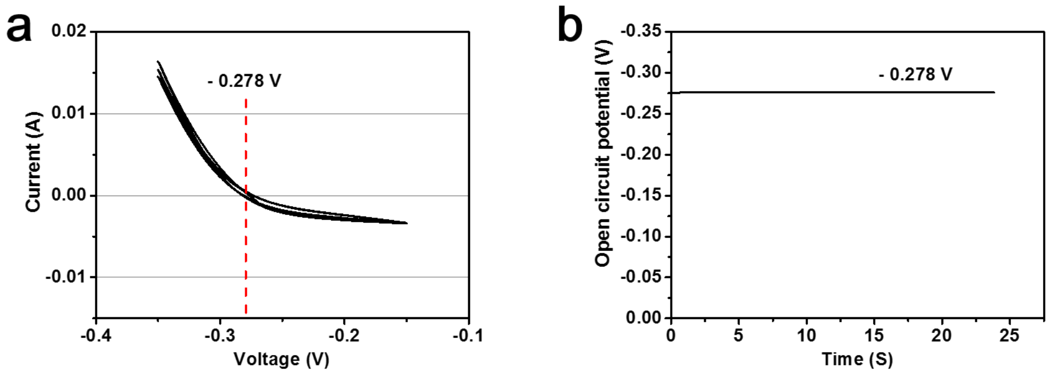

Supplementary Figure 13 | Calibration of the reference electrode. (a) Cyclic voltammetry (CV) curve of hydrogen oxidation and evolution in 0.5 M H_2SO_4; **(b)** Open circuit potential of the electrochemical cell. The saturated calomel electrode (SCE) was calibrated with respect to the reversible hydrogen electrode (RHE). The 0.5 M H_2SO_4 electrolyte was firstly saturated with ultrahigh purity hydrogen gas. A Pt net was used as the working electrode, a Pt-Rh net as the counter electrode and the SCE electrode as the reference electrode. CV was performed at a scan rate of 5 mV s^{-1}, and the average of the two potentials at which the current crossed zero was taken as the thermodynamic potential based on SCE for the H^+/H_2 reactions. The open circuit potential was also measured for this three electrode configuration to further confirm the electrode potential of SCE in 0.5 M H_2SO_4 electrolyte.

Supplementary Table 1 | HER activity comparison of the CoS|P/CNT hybrid with the most active non-noble metal based catalyst materials reported in the literature

Catalyst	η@10mA cm^{-2} (mV)	Tafel slope (mV dec^{-1})	J_0 (mA cm^{-2})	Loading (mg cm^{-2})	Reference
**CoS|P/CNT**	**48**	**55**	**1.14**	**1.6**	**This work**
	**63**	**48**	**0.83**	**0.8**	
	**67**	**49**	**0.64**	**0.4**	
CoS_2 film	232	44.6	5.40E-05	N/A	1
$CoSe_2$ film	230	42.5	5.00E-05	N/A	
$Co_{0.32}Ni_{0.68}S_2$ film	276	66.3	2.50E-04	N/A	
$Fe_{0.43}Co_{0.57}S_2$ film	192	55.9	1.60E-03	N/A	
$Co_{0.56}Ni_{0.44}Se_2$ film	252	49.7	7.10E-05	N/A	
$CoSe_2$ NPs	N/A	40	3.70E-02	0.037	
CoS_2/rGO/CNTs	143	51	6.26E-02	1.15	2
CoS_2	327	148	1.01E-02	0.280	
CoS_2/rGO	278	82	7.02E-03	0.280	
CoS_2 film	N/A	48.8	1.91E-05	N/A	3
$Fe_{0.07}Ni_{0.93}S_2$	N/A	58.7	4.69E-05	N/A	
$Fe_{0.48}Co_{0.52}S_2$	196	47.5	9.59E-04	N/A	
$Co_{0.59}Ni_{0.41}S_2$	N/A	50.4	4.76E-05	N/A	
$Fe_{0.9}Co_{0.1}S_2$/CNT	160	46	N/A	0.4	4
CoS_2 nanopyramids on Ti foil	193	72	N/A	N/A	5
CoS_2 nanowires	145	51.6	1.51E-02	1.7±0.3	6
Co_2P hollow NPs	95	45	N/A	1.0	7
CoP hollow NPs	75	50	N/A		
CoP nanowire arrays on carbon cloth	67	51	0.288	0.92	8
CoP@CNT	117	54	0.13	N/A	9
FeP NPs	50	37	0.43	1.0	10
CoS_2/P nanosheets	67	50	0.47	2.5	11
CoPS NPls	48	56	0.984	N/A	12
CoPS film	128	57	0.056	N/A	
CoPS NWs	61	75	0.554	N/A	

1T MoS_2<br>2H MoS_2	210<br>368	~40<br>75-85	N/A	N/A	[13]
Mo phosphosulfide	86	50	0.2	1.0	[14]
Li intercalated MoS_2	113	43~47	0.13~0.25	0.12	[15]
MoS_2/$CoSe_2$	68	36	0.073	0.28	[16]
MoP NPs	125	54	0.086	0.36	[17]

Supplementary Methods

Materials

$NaH_2PO_2 \cdot H_2O$ was purchased from Alfa Aesar. H_2SO_4, $KMnO_4$, absolute alcohol, $NaNO_3$, NaOH, and H_2O_2 (30% solution) were obtained from VWR Corporation. CNTs (FloTube™ 9000) were received from CNano Technology (Beijing) Ltd. Thioacetamide and cobalt acetate was purchased from Acros Organics. Nafion (5 wt%) was bought from Sigma-Aldrich. All the reagents were used as received. The water used throughout all experiments was purified through a Millipore system.

Preparation of mildly oxidized CNTs

Mildly oxidized CNTs were prepared through a modified Hummers method[18]. 1 g of multi-wall CNTs were first purified by calcination in air at 500 °C and refluxing with 40 mL of diluted hydrochloric acid (10%) to remove amorphous carbon and residual catalyst particles. The purified CNTs were washed with DI water and dried in oven at 80 °C overnight. Then, the purified CNTs were moved into a 250 mL flask and 23 mL of concentrated sulfuric acid was added. The mixture was stirred at room temperature for 12 h. After that, the flask was heated to 40 °C in an oil bath, followed by slow addition of 100 mg of $NaNO_3$. After 5 min, 4 g of $KMnO_4$ was slowly added into the suspension, keeping the reaction temperature no higher than 45 °C. The suspension was stirred for 30 min and followed by addition of 3 mL of DI water. 5 min later, another 3 mL of DI water was added. After another 5 min, 40 mL of DI water was added. 15 min later, the flask was removed from the oil bath and 140 mL of water and 30 mL of 30% H_2O_2 was added to stop the reaction. The suspension was stirred at room temperature for 15 min. It was then centrifuged and washed with 5% hydrochloride acid solution for 3 times, followed by repeated wash with DI water until pH value reached higher than 5. The final precipitate was then collected and freeze-dried.

Materials characterizations

XRD measurements were performed using a Riguku Smartlab X-ray diffractometer with Cu Kα radiation. SEM images were obtained on a Hitachi SU70 field emission SEM. Low-magnification TEM measurements were performed on a JEM 1400 (JEOL USA) with an accelerating voltage at 80 kV. High-resolution TEM imaging and STEM-EDS mapping were done with an FEI TECNAI Osiris at an accelerating voltage of 200 kV. XPS studies were performed using a Kratos Axis Ultra X-ray photoelectron spectrometer with Al Kα radiation ($h\nu$ = 1486.71 eV). Raman spectra were obtained using a confocal micro-Raman spectroscopy (LabRam HR Evolution, Horiba) with a 532 nm excitation laser.

Colorimetric comparison

2.5 mg of catalyst material was soaked into 10 mL of 0.5 M H_2SO_4 solution for 2 h. After that, the mixture was centrifuged and the supernatant was collected. 20 µL of supernatant, 5 mL of sodium acetate solution (250 g L^{-1}), and 3 mL of Nitrite R salt solution (10 g L^{-1}) were added

into a 25 mL vial. The dissolved Co^{2+} can form a red-colored complex with the nitrite R salt in acetate buffer solution.

Supplementary References

1. Kong DS, Cha JJ, Wang HT, Lee HR, Cui Y. First-row transition metal dichalcogenide catalysts for hydrogen evolution reaction. *Energy Environ Sci* **6**, 3553-3558 (2013).
2. Peng S*, et al.* Cobalt sulfide nanosheet/graphene/carbon nanotube nanocomposites as flexible electrodes for hydrogen evolution. *Angew. Chem. Int. Ed. Engl.* **53**, 12594-12599 (2014).
3. Faber MS, Lukowski MA, Ding Q, Kaiser NS, Jin S. Earth-Abundant Metal Pyrites (FeS, CoS, NiS, and Their Alloys) for Highly Efficient Hydrogen Evolution and Polysulfide Reduction Electrocatalysis. *J Phys Chem C Nanomater Interfaces* **118**, 21347-21356 (2014).
4. Wang DY*, et al.* Highly active and stable hybrid catalyst of cobalt-doped FeS_2 nanosheets-carbon nanotubes for hydrogen evolution reaction. *J. Am. Chem. Soc.* **137**, 1587-1592 (2015).
5. Zhang H*, et al.* Highly Crystallized Cubic Cattierite CoS_2 for Electrochemically Hydrogen Evolution over Wide pH Range from 0 to 14. *Electrochim. Acta* **148**, 170-174 (2014).
6. Faber MS, Dziedzic R, Lukowski MA, Kaiser NS, Ding Q, Jin S. High-performance electrocatalysis using metallic cobalt pyrite CoS_2 micro- and nanostructures. *J. Am. Chem. Soc.* **136**, 10053-10061 (2014).
7. Callejas JF, Read CG, Popczun EJ, McEnaney JM, Schaak RE. Nanostructured Co_2P Electrocatalyst for the Hydrogen Evolution Reaction and Direct Comparison with Morphologically Equivalent CoP. *Chem. Mater.* **27**, 3769-3774 (2015).
8. Tian J, Liu Q, Asiri AM, Sun X. Self-supported nanoporous cobalt phosphide nanowire arrays: an efficient 3D hydrogen-evolving cathode over the wide range of pH 0-14. *J. Am. Chem. Soc.* **136**, 7587-7590 (2014).
9. Liu Q*, et al.* Carbon Nanotubes Decorated with CoP Nanocrystals: A Highly Active Non-Noble-Metal Nanohybrid Electrocatalyst for Hydrogen Evolution. *Angew. Chem. Int. Ed.* **53**, 6710-6714 (2014).
10. Callejas JF*, et al.* Electrocatalytic and Photocatalytic Hydrogen Production from Acidic and Neutral-pH Aqueous Solutions Using Iron Phosphide Nanoparticles. *ACS Nano* **8**, 11101-11107 (2014).
11. Ouyang C*, et al.* Hierarchically Porous Ni_3S_2 Nanorod Array Foam as Highly Efficient Electrocatalyst for Hydrogen Evolution Reaction and Oxygen Evolution Reaction. *Electrochim. Acta* **174**, 297-301 (2015).
12. Caban-Acevedo M*, et al.* Efficient hydrogen evolution catalysis using ternary pyrite-type cobalt phosphosulphide. *Nat. Mater.* **14**, 1245-1251 (2015).
13. Voiry D*, et al.* Conducting MoS_2 nanosheets as catalysts for hydrogen evolution reaction. *Nano Lett.* **13**, 6222-6227 (2013).
14. Kibsgaard J, Jaramillo TF. Molybdenum Phosphosulfide: An Active, Acid-Stable, Earth-Abundant Catalyst for the Hydrogen Evolution Reaction. *Angew. Chem. Int. Ed.* **53**, 14433-14437 (2014).
15. Wang H*, et al.* Electrochemical tuning of vertically aligned MoS_2 nanofilms and its application in improving hydrogen evolution reaction. *Proc. Natl. Acad. Sci. U. S. A.* **110**, 19701-19706 (2013).
16. Gao MR*, et al.* An efficient molybdenum disulfide/cobalt diselenide hybrid catalyst for electrochemical hydrogen generation. *Nat Commun* **6**, 5982 (2015).
17. Xing Z, Liu Q, Asiri AM, Sun X. Closely interconnected network of molybdenum phosphide nanoparticles: a highly efficient electrocatalyst for generating hydrogen from water. *Adv. Mater.* **26**, 5702-5707 (2014).
18. Liang Y*, et al.* Oxygen reduction electrocatalyst based on strongly coupled cobalt oxide nanocrystals and carbon nanotubes. *J Am Chem Soc* **134**, 15849-15857 (2012).
